# Establishment of Efficient CRISPR-Cas9 PEG-Mediated DNA-Free Genome Editing Through Ribonucleoproteins Method in Hexaploid Sweetpotato (*Ipomoea batatas* L. (Lam)) Targeting the *EIF*-*4E* Genes

**DOI:** 10.3390/plants15030447

**Published:** 2026-02-01

**Authors:** Adrianne P. A. Brown, Marceline Egnin, Foaziatu Bukari, Inocent Paulin Ritte, Gregory C. Bernard

**Affiliations:** 1 Animal and Plant Health Inspection Services (APHIS) Brasilia Embaixada dos Estados Unidos da America, SES-Avenue. das Nações, Brasilia 07457, Brazil; 2 Plant Biotech and Genomics Research Lab, Department of Agricultural and Environmental Sciences, College of Agriculture, Environmental and Nutritional Sciences (CAENS), Tuskegee University, Tuskegee, AL 36088, USAiritte8222@tuskegee.edu (I.P.R.); gbernard@tuskegee.edu (G.C.B.)

**Keywords:** sweetpotato *Ipomoea batatas*, CRISPR/Cas9 (CRISPR-associated protein 9), genome editing, CRISPR-Cas9 ribonucleoprotein (RNPs) complexes, PEG-mediated, protoplast transfection, eukaryotic translation initiation host susceptibility factor 4E, transgene-free gene editing, polyploid and vegetatively propagated crops

## Abstract

CRISPR-Cas9 technology has opened new perspectives in genome editing of clonally, asexually propagated and polyploid plants by enabling multiple allelic gene edits. Traditional *Agrobacterium*- and particle bombardment-mediated transformations, which rely on integration of gene-editing transgene cassettes, have been efficiently applied to several plants; however, concerns about the acceptability of resultant edited transgenic genotypes make these methods less attractive for vegetatively propagated crops. We leveraged and optimized the CRISPR-Cas9/sgRNA-RNPs system for delivery into protoplasts of the hexaploid sweetpotato cultivar PI-318846, targeting eukaryotic translation initiation factor isoform *4E* genes to enhance resistance to SPFMV potyviruses. To evaluate the efficiency of pre-assembled Cas9/sgRNA-RNP in sweetpotato transfection, single guide RNAs were designed to target putative host susceptibility genes: *IbeIF4E*, *IbeIF(iso)4E*, and *IbCBP*. Freshly isolated leaf protoplasts were subjected to CRISPR-CAS9-RNP PEG-mediated transfection under different parameters. Sweetpotato regenerants screened using PCR-RE-T7 assay, sequencing, and Inference CRISPR Edit analyses of target-site amplicons revealed the most efficient editing conditions utilizing 25% PEG with a 3:1 (15 µg:45 µg) ratio of Cas9/sgRNA-RNP for 25 min and 48 h incubation period. Different allelic InDels were obtained with editing efficiencies of 10–20% in regenerated plantlets, demonstrating that PEG-mediated CRISPR-RNP transfection system is key for advancing DNA-free editing tools in polyploid and vegetatively propagated crops.

## 1. Introduction

New breeding technologies, particularly genome editing, have emerged as a powerful alternative for crop improvement; in 2020, Jennifer Doudna and Emmanuelle Charpentier received the Nobel Prize in Chemistry for developing a new genome-editing tool derived from *Streptococcus pyogenes* (Sp.) [1,2]. Since the inception of SpCas9 genome editing [2], delivery of clustered regularly interspaced short palindromic repeat (CRISPR) components and their associated protein-9 (Cas9, Cas endonucleases) (CRISPR/Cas9) complexed with single guide RNA (sgRNAs), in plants has relied primarily on *Agrobacterium*-mediated transformation and particle bombardment, with increasing improvements to date [3,4,5,6,7]. While effective, these methods often result in random integration of Cas9/sgRNA expression cassettes, increasing the risk of off-target mutations, genome impairment, and constitutive transgene expression, leading to key regulatory concerns worldwide [8,9], particularly for vegetatively propagated crops that cannot eliminate transgene through conventional breeding and segregation [7]. Although some of these concerns can potentially be addressed through conventional breeding, such approaches are labor-intensive and often impractical for crops that are propagated vegetatively, such as sweetpotato [7,10]. To mitigate these limitations, investigators have performed plasmid-independent CRISPR/Cas9 gene editing [11,12,13,14] using DNA-free ribonucleoprotein complexes (RNPs) delivered through electroporation or polyethylene glycol (PEG) ensuring direct editing of the genetic material without transgene integration [13,14]. PEG-mediated CRISPR-Cas9-RNPs protoplast transfection protocols have been established for many plants, including model species such as *Arabidopsis* and tobacco; staple crops such as rice, corn, soybean, and wheat; ecologically important tree species; and roots and tubers, including potato, cassava, and sweetpotato, with demonstrated recovery of transgene-free plantlets [13,14,15,16,17,18,19,20,21,22]. During transfection, the RNP complex shuttles through the plasma and nuclear membranes to perform sequence-specific editing and is subsequently degraded by endogenous nucleases and proteases [23]. Using this procedure to perform editing has shown improvements compared with other methods by mitigating risks for random integration, reducing off-targeting effects, and eliminating key regulatory concerns [24].

Sweetpotato (*Ipomoea batatas* L. (Lam)) is a globally significant root crop valued for its nutritional density contributing high levels of beta-carotene, anthocyanins, dietary fiber, essential minerals and proteins [25,26,27,28,29,30]. Sweetpotato ranks as the world’s third most important root and tuber crop after potato and cassava [31], with global production exceeding 91.8 million metric tons annually [32]. China is the leading producer, while the United States is the largest exporter, with exports valued at USD 198.8 million [33,34].

Despite its nutritional value and resilience in marginal environments, sweetpotato production has been declining in recent years, due to its vegetative propagation system [35], abiotic stresses [28,36], virus accumulation in vegetatively propagated planting material [37], and widespread biotic stressors—including insects, weeds, fungal pathogens, sweetpotato weevils (*Cylas formicarius*), root-knot nematodes (*Meloidogyne* spp.), *Ceratocystis fimbriata*, and numerous viruses that continue to reduce yield and root quality across production regions [38,39].

Sweetpotato improvement is hindered by fundamental genetic and reproductive bottlenecks: severe cross incompatibility, poor seed setting, self-incompatibility, high heterozygosity, and a highly complex hexaploid allopolyploid genome (2n = 6x = 90) [40,41]. These factors make conventional breeding labor intensive, slow, and often unfeasible for vegetatively propagated crops such as sweetpotato. Although the recent high-quality genome assembly of the hexaploid cultivar ‘Tanzania’ [42] now facilitates the characterization of homologous alleles for functional analyses and CRISPR/Cas9-mediated editing, efficient, genotype-independent transformation and reliable plant regeneration systems remain critically limited.

The integrative nature of CRISPR–Cas ribonucleoproteins (RNPs) has opened transformative new avenues for genome engineering in crops that are otherwise difficult to breed, especially those with multiple chromosome sets, such as sweetpotato [10,43]. CRISPR-RNP delivery offers several advantages, including transgene-free editing, reduced off-target effects, and applicability to recalcitrant species, which align with the unique biological challenges imposed by the sweetpotato allopolyploidy genome. Given the complexity of the hexaploid genome, modifying agronomically important traits will require precise targeting across multiple homologous gene copies, underscoring the need for a robust, reproducible editing and regeneration pipeline.

To strengthen sweetpotato viability and productivity, genetic engineering tools—including CRISPR, viral vectors, and transgenic approaches—have been used to modify genes associated with disease susceptibility, starch quality, abiotic stress tolerance, and resistance to viruses, nematodes, herbicides, and insect pests [5,10,41,44,45,46,47,48,49,50]. However, continued progress requires genome-editing systems optimized for the sweetpotato polyploid genome, together with efficient protoplast regeneration systems capable of producing edited lines suitable for both small-scale and commercial farming systems.

Among trait-specific constraints, sweetpotato feathery mottle virus (SPFMV), sweetpotato chlorotic stunt virus (SPCSV), and their synergistic complex, sweetpotato virus disease (SPVD), represent the most extensively studied virus systems in sweetpotatoes [5,51,52]. SPVD results from co-infection by SPCSV, a whitefly-transmitted crinivirus, and SPFMV, an aphid-transmitted potyvirus, whose interaction markedly enhances symptom severity and viral accumulation [46,53]. These viruses possess compact RNA genomes and rely entirely on host translation machinery for replication, making host translation-associated factors central to virus–host interaction studies [54,55].

Across plant species, the susceptibility to potyviruses has been associated with conserved translation-related genes, including eukaryotic initiation factor *4E (eIF4E)*, its isoform *eIF(iso)4E*, and cap-binding protein (CBP) [56,57,58,59]. Because these genes are essential for cellular translation and conserved across crops, they have been widely used as functional genomic targets and proof-of-concept loci for CRISPR/Cas-mediated genome editing in *Arabidopsis*, tobacco, wheat, rice, melon, tomato, potato, cassava, and sweetpotato [17,60,61,62,63,64,65,66].

In sweetpotatoes, the genetic architecture of these loci remains incompletely resolved. Diallelic crossing experiments suggest inheritance in a tetrasomic or hexasomic manner, reflecting the complexity of the hexaploid genome [42,67,68]. Recent work by [40] identified and characterized members of the *eIF4E* gene family in sweetpotato—including *IbeIF4E*, *IbeIF(iso)4E*, and *IbCBP* (GenBank OP273667–OP273690)—defining their exon–intron structures and coding sequences across multiple cultivars, with several allelic homeologs; thus, providing a well-defined molecular targets for genome-editing studies.

Our research program has established robust sweetpotato biotechnology systems, including embryogenic-callus induction from leaf explants and protoplasts, somatic embryo-derived plant regeneration, and transgenic plant development [48]. We developed an efficient nurse-leaf protoplast culture system that enables whole-plant regeneration and CRISPR/Cas9 genome editing, achieving targeted *IbPDS* editing and regeneration of edited plantlets in hexaploid sweetpotato using PEG-mediated plasmid DNA delivery of Cas9/sgRNA complexes [16,69]. Building on this foundation, the current study aims to develop an efficient transgene-free CRISPR/Cas9 ribonucleoprotein (RNP) system in sweetpotato protoplasts targeting *eIF(iso)4E* gene across multiple cultivars, providing a future platform for genome editing in crops with complex polyploid genomes.

## 2. Results and Discussion

### 2.1. Evaluation of Target-Site Selection and Single-Guide RNA Design

**Figure 1:**
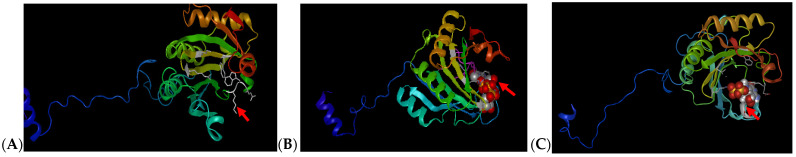
Modeling of selected eIF4E Proteins Secondary and Tertiary Structures based on computational prediction software. (**A**): modeling of the sweetpotato *eIF4E* containing a total of nine alpha helices and eight beta-pleated sheets, with a binding pocket which is larger than average. (**B**): modeling of the sweetpotato eIF(iso)4E protein with a total of six alpha helices and eight beta-pleated sheets. (**C**): modeling of the sweetpotato *IbCBP* protein with a total of six alpha helices and eight beta-pleated sheets. Red arrows are indicative of ligand binding sites. Prediction was based on predicted homology of Chain A and Chain B of wheat eIF4E as described by [71].

**Figure 2:**
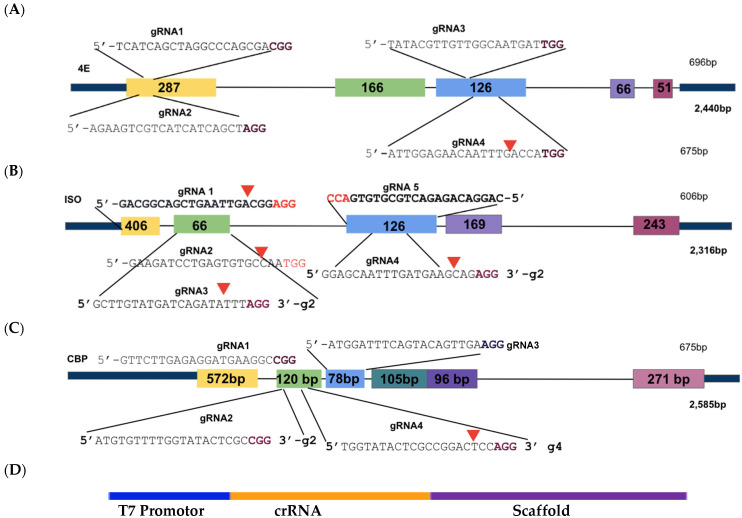
Illustration of sgRNA location as CRISPR/Cas9 target sites within the *4E* gene sequences. (**A**): *IbeIF4E*; (**B**): *IbeIF(iso)4E*; (**C**): *IbCBP*. Nucleotide sequences in black indicate the crRNA sequence, accompanied with PAM sites indicated in purple. The first three exons of each gene were targeted to ensure gene Knock-Out. Based on the Cas version (SpCas9), protospacer adjacent PAM (NGG) sites were identified 20 bp upstream (crRNA). (**D**): Schematic of the cassette with T7 Promoter (for guide-it in vitro assay only), and crRNA and Scaffold template (tracrRNA). CRISPR/Cas9 target sites within *IbeIF(iso)4E* herein were utilized for CRISPR-Cas9-RNA transfection and regeneration of sweetpotato protoplast.

Previous cloning, sequencing and characterization analysis performed on sweetpotato *eIF4E* genes within four different cultivars (Beauregard, Resisto, D-3, and Jewel) have revealed many single-nucleotide polymorphisms (SNPs) within the coding regions, which can be a limiting factor in developing common target sites to perform site-specific editing amongst various varieties [40,60]. To further validate protein characterization and assess potential gRNA target sites, homology modeling was performed to obtain secondary and tertiary structure predictions for each protein. Secondary structure prediction studies performed in *IbeIF4E* showed eight alpha helices and eight ß-pleated sheets (Figure 1A, Table 1). In *IbeIF(iso)4E*, six alpha helices and eight ß-pleated sheets were obtained (Figure 1B, Table 1). CBP showed a total of six alpha helices and eight beta-pleated sheets (Figure 1C). In comparing secondary structures against those of other species, the *IbeIF4E* protein appears to contain the same amount of ß-pleated sheet found in wheat *eIF4E*. However, where wheat *eIF4E* contains four alpha helices, sweetpotatoes contain a total of nine (Table 1). In *IbeIF(iso)4E*, prediction analysis identified two additional alpha helices as compared to four alpha helices discovered in *eIF(iso)4E* of tomato [66]. The *IbCBP* possesses eight ß-sheets and a total of three alpha helices compared to *E. coli* CBP [17]. Knowledge obtained from earlier research shows that alpha helices are often associated with being used for protein–DNA interaction, which should be expected in proteins such as *eIF4E* that bind to mRNA caps for translation initiation [40,70]. These sequences and secondary structures obtained from this analysis were subsequently utilized to design the sgRNA and mutation target-site (Figure 2, Table 2). Figure 1 shows 3D illustrations of predicted ribbon tertiary structures. The red arrows indicate ligand binding pockets.

### 2.2. Analysis of In Vitro Digestion Assay

**Figure 3:**
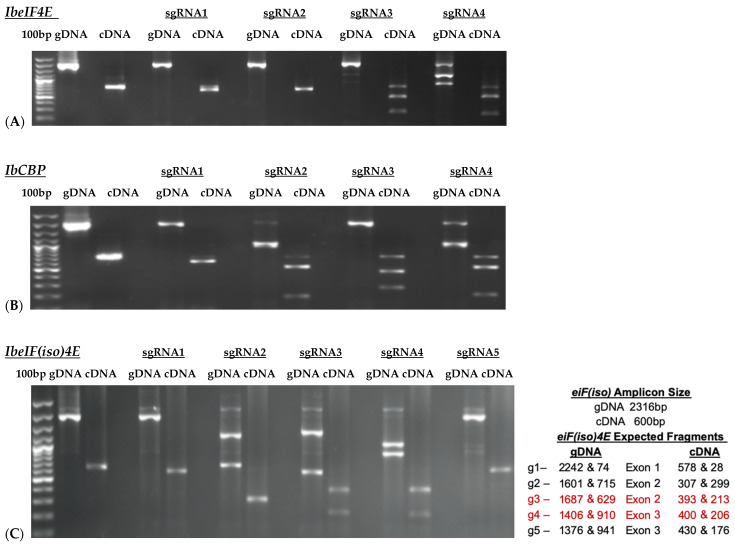
Results of InVitro Guide-IT Digestion assay of sgRNA screening of sweetpotato eIF4E genes. sgRNA sequences were in vitro transcribed and evaluated against: (**A**) *IbeIF4E*, (**B**) *IbCBP* and (**C**) *IbeIF(iso)4E* genes gDNA and cDNA amplicons from D-3. The first lane in each gel shows the 100 bp ladder. The two lanes following the ladder show un-cleaved genomic DNA and cDNA control fragments. The remaining lanes are the gDNA and cDNA amplicons that were treated with Cas9/sgRNA complex. The red text highlights the most efficient single guide RNA tested.

**Figure 4:**
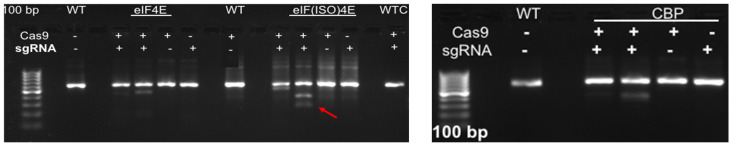
gRNA efficiency utilizing the Guide-it-In Vitro transcription and screening system. The 600 bp cDNA amplicons of *IbeIF4E*, *IbeIF(iso)4E* and *IbCBP* were subjected to either Cas or sgRNA or the complex to confirm its efficiency. The following cleaved fragments were obtained for *eif4E* g1-322/279 and g2-450/151, *eif(iso)4E* g3-517/95 and g4-376/236, CBP g2-339/270 and g3-331/278. (+) and (-) signs signify with and without Cas9 or sgRNA treatments. Red arrow indicate resulting edited fragments.

Based on the cloned sequences and predicted protein structures, sgRNAs were designed (Figure 2; Table 2), synthesized, and subjected to in vitro cleavage activity. Fifty ng/µL of in vitro transcribed sgRNA pre-assembled with 250 ng of Cas9 nuclease were added to 250 ng of PCR-amplified target gDNA or cDNA samples according to protocol recommendations. PCR-amplified genomic DNA fragments yield larger fragment sizes than cDNA samples, which amplify the ~600 bp Full-length (FL) sequence of each target. Out of the four gRNAs designed to target *IbeIF4E*, two (guide 3 and guide 4) were shown to cleave gDNA and cDNA amplicons, which revealed variations in their cleavage efficiency (Figure 3 and Figure 4). The gRNA2, designed to target *IbCBP*, showed the highest cleavage activity (Figure 3B and Figure 4). Of the five gRNAs designed to target *IbeIF(iso)4E*, three were found to cleave both the gDNA and FL-cDNA amplicons (guide 2, 3, and 4) (Figure 3C). The following sgRNAs were also tested in cultivars Beauregard and Resisto; which demonstrated efficient cleavage activity. To develop an optimized protocol for efficient CRISPR-Cas-RNP edits in sweetpotatoes, *IbeIF(iso)4E sgRNA 4* (Figure 2C and Figure 4) was selected for synthesis as RNA-oligonucleotide molecules for further protoplast transfection experiments using the highly regenerative sweetpotato cultivar D-3 (NZ-196, PI-318846).

### 2.3. Establishment and T7 Mutation Detection of Sweetpotato RNP PEG-Mediated Transfection System

We leveraged a previously established pipeline for sweetpotato protoplast isolation, nurse culture, and PEG-mediated delivery that was validated using plasmid DNA–based sgRNAs construct and CRISPR/Cas9 editing targeting the phytoene desaturase gene (*IbPDS*) in hexaploid sweetpotato protoplasts [16]. However, in this work of CRISPR-Cas9 ribonucleoprotein (RNP)-based protocol for DNA-free genome editing in sweetpotato, a putative host susceptibility factor, eukaryotic translation initiation factor (iso) 4E, was targeted using a purified recombinant Cas9 protein pre-assembled with a synthesized RNA-oligonucleotide sgRNA molecule by IDT.

### 2.4. Evaluation of Cas9/sgRNA and PEG Ratios

**Figure 5:**
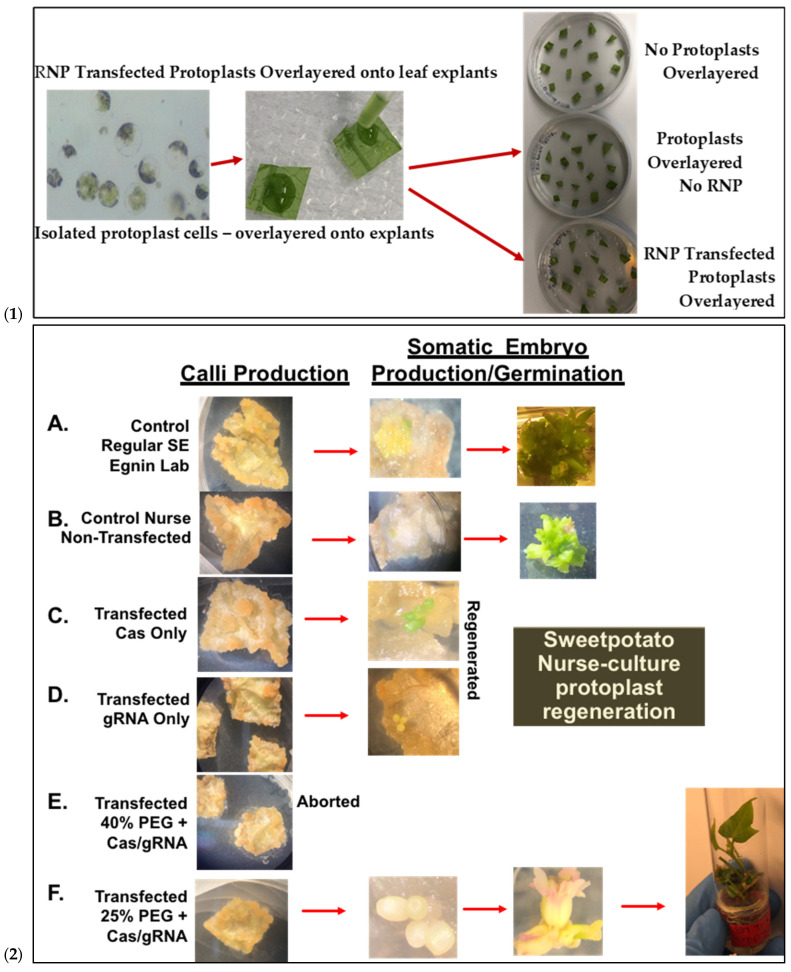
Sweetpotato nurse-culture protoplast regeneration following PEG-mediated delivery of CRISPR gene-editing reagents. (**1**): Isolated protoplasts over-layered onto plated leaf explants for Nurse-culture embryogenic regeneration after 72 h post-transfection and co-cultivated with Cas9-*IbeIF(iso)4E*-RNP. (**2**): Integrated sweetpotato somatic embryogenesis callus induction and subsequent embryo production and plantlet regeneration utilizing in-house protocol. The resulting putative gene-edited plantlet lines are screened for mutation. (**2A**): Control Regular leaf explant SE (Egnin Lab); (**2B**): Control non-transfected and nursed with 91% embryogenic response and 100% plantlets; (**2C**): Transfected Cas Only and nursed regenerated; (**2D**): transfected gRNA only; (**2E**): transfected 40%PEG-Cas/gRNA-RNP, all aborted; (**2F**): transfected 25%PEG + Cas/gRNA-RNP *IbeIF(iso)4E* with 30% of transfected samples developing embryo clusters (3–10 embryos/cluster) resulting in 12 germinated into full normal putative edited plantlets.

**Figure 6:**
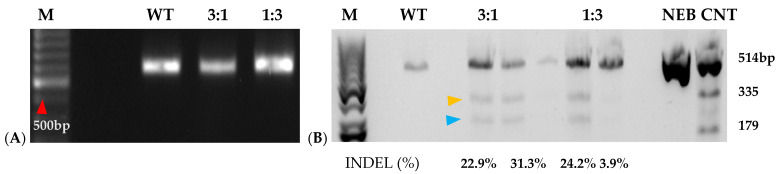
Mutation detection on targeted eIF(iso)4E guide 4 by PCR-RE of T7 heteroduplex digestion of WT and RNP-transfected sweetpotato protoplasts. (**A**): PCR amplification of ~600 bp *IbeIF(iso)4E* gene target-site fragment of protoplasts isolated from PI-318846 (D-3) and its WT, 3:1 (45 µg:15 µg), and 1:3 (15 µg:45 µg) Cas9: gRNA ratio in transfection. (**B**): Mutagenesis assessment of *eIF(iso)4E* in sweetpotato protoplasts analyzed by T7 mutation detection assay. WT indicates normal non-transfected. WT control had 100%, while the transfected samples were mixed in a ratio of 50% transfected sample with 50% control. Yellow arrow = 335 bp and blue arrow = 277 bp cleavages. NEB CNT represents the New England Biolabs (Boston, MA, USA) mutation detection T7 Kit positive control; M indicates 100bp DNA ladder (New England Biolabs) with the red arrow pointing to the 500 bp fragment; NEB, CNT, New England Biolabs mutation detection kit positive control undigested and digested.

Prior to transfection, protoplast isolation established from [16] was used to collect yields of up to 3.82 × 10^6^ from D-3 (Figure 5(1)). Integrity was assessed before transfection to ensure cell viability, which ranged from 40 to 50 develop a CRISPR RNP PEG-mediated sweetpotato protoplast transfection protocol, modifications were generated based on RNP protocols established from *Arabidopsis*, grape, apple, and maize [19,20]. Following cell viability tests, protoplasts were adjusted to 1 × 10^5^ and mixed with RNP complexes targeting *IbEIF(iso)4E*, then incubated with 25% or 40% PEG for 25 min, then washed with W5 solution. Following a two-day protoplast incubation period, protoplasts were aliquoted for gDNA extraction and for co-cultivation onto four six-week old sweetpotato nurse-feeder explants and observed throughout embryogenesis stages (Figure 5(2)). Interestingly, during our observations, opaque scabrous calli were detected on explants co-cultivated with protoplasts transfected with 40% PEG solution, which could not regenerate into full plantlets and were discarded (Figure 5(2E); Table 3). PCR products for mutation detection were amplified from transfected protoplast genomic DNA using the primers “eIF(iso)4E 2.3-FR” and “eIF(iso)4E 2.3-RV” (Figure 6). T7 endonuclease I (T7E1) mismatch assays of protoplasts transfected using 25% PEG and Cas9:sgRNA ratios of 3:1 (45 µg:15 µg) or 1:3 (15 µg:45 µg) indicated successful editing at the eIF(iso)4E g4 target site, with estimated mutation efficiencies of 22.9% and 24.2%, respectively, relative to the WT amplicon (Figure 6). The results of this investigation align with previous reports of PEG-mediated RNP delivery into protoplasts of *A. thaliana*, tobacco, maize, and rice [20]. To ensure CRISPR/Cas9-RNPs can perform endogenous gene editing, generate DSBs at the target site and repair them via NHEJ, protoplast cells were regenerated into plantlets. Out of 96 transfected protoplasts and nurse-culture experiments from D-3, 30% of the transfected protoplasts and nurse culture developed somatic embryo clusters. All the 40% PEG-transfected explants were aborted. In total, 50% of control nurse protoplasts developed embryo clusters (Figure 5(2)).

### 2.5. Sequencing Confirmation of RNP Delivery Targeting Sweetpotato eIF(iso)4E

**Figure 7:**
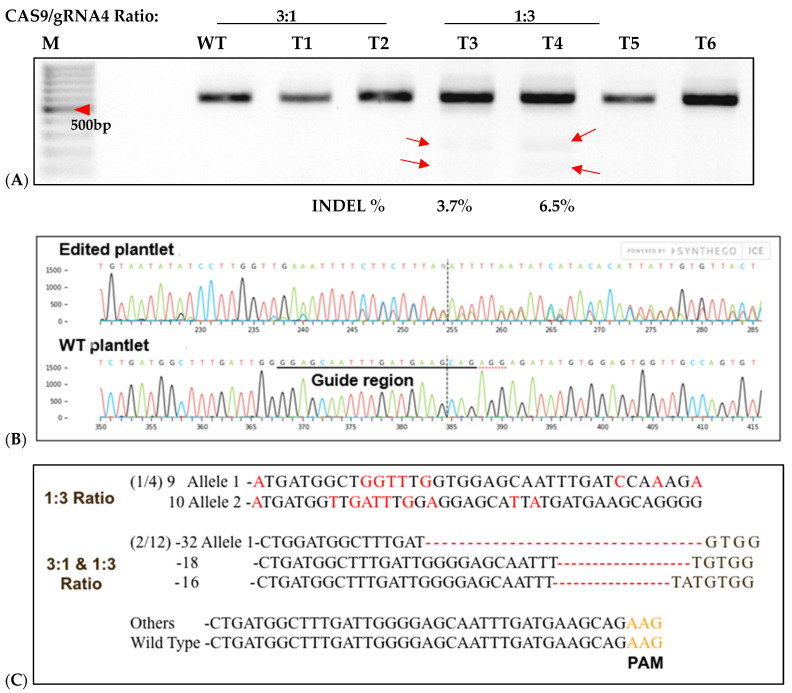
T7-Endonuclease and Sanger sequencing detection of targeted mutagenesis of *IbeIF(iso)4E* in six of 12 regenerated sweetpotato nursed protoplasts. (**A**): Mutation detection on targeted *eIF(iso)4E* by PCR-RE T7 mutagenesis of *IbeIF(iso)4E* in six of 12 regenerated sweetpotato nursed protoplasts into plantlets. Purified recombinant Cas9 and in vitro transcribed gRNA were pre-assembled in 3:1 and 1:3 ratios and transfected into sweetpotato protoplast of cv. D-3. DNA was isolated and utilized to amplify a ~600 bp fragment using ‘eIF(iso)4E 2.3-FR’ and eIF(iso)4e-RV primers. Target sites were amplified, assessed and hetero-duplexed for T7-E mutation detection. Mutation detection results were analyzed using high-resolution agarose. M: 100 bp DNA ladder; Lanes WT: untransformed protoplast regenerants. The red arrows in the figure indicate digestion of transfected regenerants. (**B**): Sequencing detection of targeted mutagenesis from chromatogram traces revealed deletions ranging from −16 to −32 bp, respectively, in both Cas9/sgRNA ratios. Of the 12 plants regenerated plantlets and (8 3:1) and (4 1:3), three possessed edits within the target-region (3.1%) mutation rate. (**C**): Mutation populations detected in 3:1 and 1:3 mutation samples (red letters). Red dashes within the sequences indicate space for alignment purposes and numbers with negative signs signify nucleotide deletion.

**Figure 8:**
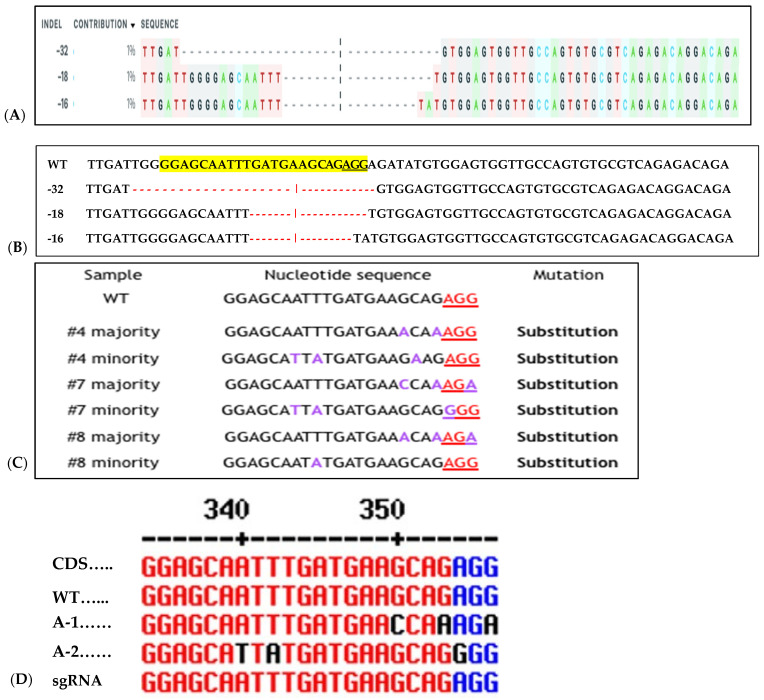
Sequence detection of RNP-induced mutations in six of 12 regenerated sweetpotato. Examples of mutation types identified in *IbeIF(iso)4E* using CRISPR/Cas9 RNPs. (**A**): relative contribution of each sequence (normalized); (**B**): WT indicates the sequenced wild-type control sample. In the WT sample, the PAM site is underlined in black. Red dashes indicate deletions detected in target site. Substituted Targeted Mutagenesis of *IbeIF(iso)4E* (**C**): Substitution mutations identified in RNP-transfected protoplast regenerated plantlets. The PAM sequences are displayed in blue and inserted nucleotides are shown in black. (**D**): CDS, Coding sequence, WT, wild-type A-1 allelic form 1, A-2 allelic form 2 and sgRNA is the original gRNA target sequence. Indication of three mutant alleles were identified by Sanger sequencing (3:1) Cas9:sgRNA ratio. The black letters indicate substitution; blue nucleotides indicate PAM sequences within the target site. Black and red dashes within the sequences indicate space for alignment purposes. WT indicates wild type control.

To assess CRISPR-Cas9 RNP-induced DSBs at the *IbeIF(iso)4E* target site in regenerated plantlets, gDNA was isolated from putative edited regenerated plantlets and control WT, subjected to PCR to amplify the target region for T7 mutation analysis and sequencing. Of the 96 transfected explants, a total of 12 embryos regenerated into plantlets, with a 12.6% regeneration rate (6 plantlets were recovered from 3:1 transfected samples and 4 plantlets were collected from 1:3 transfected samples) (Figure 7). One regenerated plantlet was maintained for samples transfected with sgRNA only and Cas9 only. The 600 bp target region from regenerants was digested with T7E1, and the expected bands (335 bp and 227 bp approximately) were observed in all five edited lines (Figure 7). The wild-type plants did not show these bands, confirmed editing in six lines. Sequencing of the complete amplicon followed by analysis with Mixed Sequence Reader, which interprets overlapping chromatogram peaks to identify base-level changes, revealed nucleotide substitutions. In contrast, Synthego’s ICE analysis, which deconvolutes mixed Sanger traces to estimate indel profiles, detected deletions ranging from 16 to 32 bp. Based on somatic embryogenesis regeneration results, T7 mutation detection screening of regenerated plantlets using 25% PEG with 3:1 and 1:3 Cas9/sgRNA ratios showed that the targeted eIF(iso)4E gene by CRISPR/CAS9 RNP resulted in multi-allelic InDelS in hexaploid sweetpotato. Mutation detection and sequencing of the *IbeIF(iso)4E* gene amplicon and mining of the chromatogram in multiple base calls revealed indels at various locations near the sgRNA target site (Figure 7 and Figure 8), generating premature stop codons in the eIF4E protein [60]. The three allelic forms of *IbeIF(iso)4E* identified with the gRNAs were designed to target a conserved region in exon 3, inactivating the gene function. Three mutant alleles were identified by sequencing (3:1) Cas9:sgRNA ratio in three plants. The maximum multiple allelic edits resolved was three (Figure 7C) in three of the plantlets out of the 12 recovered plantlets, correlating to the three allelic forms of the *IbeIF(iso)4E* gene. Biallelic edits were obtained in 2 plantlets (Figure 7C and Figure 8C,D), and mono-allelic edits in two others (Figure 7 and Figure 8). CRISPR/Cas9 RNP has significant potential for multi-allelic gene editing, with implications for future sweetpotato breeding with SPFMV resistance. Based on the T7 mutation detection analysis described by [16] during CRISPR targeting of *IbPDS* in sweetpotato, the most effective approach for detecting mutations in regenerated, transfected samples was to generate heteroduplexes by mixing edited and wild-type PCR products. This heteroduplexing step enhances T7EI sensitivity by allowing mismatches between edited and unedited strands to be more efficiently recognized and cleaved. Thus, we performed a T7 mutation detection analysis using 100% transfected samples, and indels were detected in two of the total 12 samples screened at the expected position with frequencies that ranged from 3.5% to 6.5% in both 3:1 and 1:3 Cas9/gRNA ratios, respectively (Figure 7B,C). One caveat of using T7 mutation detection analysis is the classification of differences between mono-allelic and biallelic mutations. The gDNA was utilized to amplify a 600 bp *IbeIF(iso)4E* target site. Following PCR, reaction mixtures were PCR-purified and sequenced by GENEWIZ (South Plainfield, NJ) to mine chromatogram traces for mutations using the Synthego Inference of CRISPR Edits (ICE) online web tool (https://ice.editco.bio/#/). The Ab1. Files uploaded to the ICE platform revealed up to three allelic forms with deletions within the target site, compared with the non-transfected wild type, in both 1:3 and 3:1 Cas9/sgRNA transfections (Figure 8) Similar results have been identified in other crops such as grape and apple protoplasts, *Brassica* species, peas, and *Arabidopsis thaliana* in which indel frequencies did not significantly alter between the two 20 µg and 60 µg Cas9 RNP concentrations investigated [14,15,19,20]. Sequencing results of regenerated plantlets identified nucleotide substitutions in sample T5 that did not exhibit mutations on the resolved gel from T7 mutation detection analysis in samples transfected with 1:3 Cas9 RNP ratios (Figure 7 and Figure 8). So far, there have been no phenotypic variations in comparison to the wild type.

## 3. Materials and Methods

### 3.1. Plant Material

Sweetpotato, *Ipomoea batatas* L. (Lam), cultivar NZ-196 (PI-318846, known as D-3) nodal cuttings were obtained from the Regional Plant Genetic Resource Conservation Unit of USDA (Griffin, GA, USA) and maintained as in vitro grown plantlets at Tuskegee University Plant Biotech and Genomics Research Lab [48]. Nodal cuttings were initiated into plantlets on multiplication media (MM: MS, 30 g/L sucrose and 5 mg/L gibberellic acid (GA3) at pH 5.8 [48,72,73,74]. Plantlets were micro-propagated every 3–4 weeks by cutting two-node stem segments, placed vertically on MM in Magenta GA-7 vessels (Magenta corporation, Chicago, IL, USA) and kept under a 16 h light (50 μmol m^−2^ s^−1^) and 8 h dark photoperiod at 26 °C ± 1 °C. The apical leaves, positions 1 to 5, were utilized for protoplast isolation and as nurse explants in regeneration. The NZ-196 or PI-318846, known as D-3 is a sweetpotato cultivar that is highly regenerative in tissue culture and originated from the Timor Islands Indonesia [72,73].

### 3.2. Target-Site Selection and Single-Guide RNA Design

The sweetpotato *eIF’s* within the *4E* family gene sequences (*IbeIF4E*, *IbeIF(iso)4E*, *IbCBP*) with their multiple allelic forms, were obtained from [40,60] previously cloned and sequenced data within sweetpotato genome (Genbank OP273667-OP273690). The *eIFs* within the 4E family have been identified to play many roles outside of protein synthesis; one of the most significant has been their role in potyvirus survival. Natural mutations in these host factors show recessive resistance to many potyviruses. Protein modeling analysis was performed to aid in designing effective sgRNA in three allelic forms of *IbeIF(iso)4E* that target a conserved region in exon 3 to inactivate gene function. For this study, thirteen sgRNAs were designed in total: four targeting conserved regions of *IbeIF4E*, five targeting conserved regions of *IbeIF(iso)4E*, and four targeting conserved regions within *IbCBP* (Figure 2, Table 1 and Table 4). The sgRNAs suitable target sites were selected based on the following criteria: location within conserved regions of the first three exons of each gene to ensure functional inactivation (Figure 1); identification of favorable PAM sites specific to SpCas9; counting 20 bp upstream from each PAM site to determine the 5′ sgRNA start site; maintaining a GC content of 40–60%; rich, ensuring nucleotide repeats did not exceed 2 bp; and screening all selected designs for off-target sites using the sweetpotato genomics resource database (http://sweetpotato.uga.edu/. Accessed on 22 May 2022). Following manual design, all sgRNA sequences were confirmed using gRNA software CHOPCHOP (https://chopchop.cbu.uib.no/) [75], and sent to Azenta, formerly GENEWIZ (South Plainfield, NJ, USA) for synthesis of sgRNA as well as its transcript form (Table 2).

### 3.3. In Vitro sgRNA Cleavage Assay

To evaluate sgRNA cutting efficiency prior to protoplast transfection and plant regeneration, each sgRNA was in vitro-transcribed, purified to remove DNA contaminants, and assessed for cleavage activity using purified recombinant Cas9 protein (160 kDa) provided in the Guide-it™ Complete sgRNA Screening System (Takara Bio USA, Inc., San Jose, CA, USA). For invitro transcription, DNA templates were generated by designing forward primers containing the 28 bp T7 promoter sequence, the 20 bp sgRNA target listed in Table 4, and a 21 bp segment of the tracrRNA scaffold (Integrated DNA Technologies, Coralville, IA, USA). Additional non-target nucleotides were included at the 5′ end (shown in red in Table 4) to reduce exonuclease-mediated degradation and improve transcription efficiency. Following in vitro transcription, sgRNA samples were quantified for cleavage testing using sweetpotato leaf-derived double-stranded template DNA. To generate cleavage template, PCR was performed, with reagents provided in the Complete sgRNA Screening System Kit, under the following conditions: 98 °C for 2 min, 30 cycles of 98 °C 1 min and 60 °C for 1 min, 68 °C for 1 min with a final holding temperature of 4 °C. Primers were designed to amplify both the full-length gene and a 600 bp fragment containing the sgRNA target site, which is asymmetrically positioned within the amplicon (Table 5). In vitro cleavage reactions were performed according to the manufacturer’s instructions, and digestion products were resolved on a 2% agarose gel. sgRNAs that demonstrated efficient cleavage of both genomic DNA- and cDNA-derived amplicons were selected and subsequently synthesized as RNA oligonucleotides by Integrated DNA Technologies (IDT). The crRNAs were designed to target the gene’s third exon using the CRISPR-Cas9 Guide RNA Design Checker platform (IDT, Coralville, IA, USA). Purified recombinant Cas9 protein (160 kDa) was obtained commercially from IDT. For sweetpotato RNP mutagenesis protocol development integrated with protoplast embryogenic-callus induction and regeneration, efficient sgRNAs were synthesized by IDT as RNA-oligonucleotide molecules and utilized in subsequent experiments.

### 3.4. Sweetpotato Leaf Protoplast Isolation

**Figure 9:**
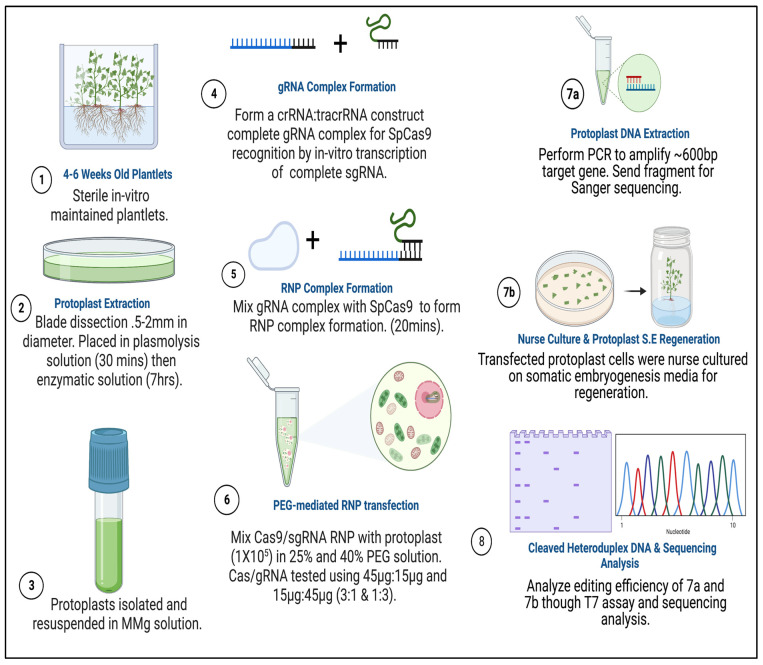
Experimental design and methodological approach of CRISPR/Cas9 RNP mutagenesis integrated with embryogenic-callus induction and regeneration in sweetpotatoes. (**1**) Plantlets were grown in GA−7 vessels for 4–6 weeks in a controlled environment. (**2**) Protoplast extraction takes place using 4–6 weeks old plantlets and is submerged in enzyme digestion solution for up to 7 h. (**3**) Protoplast are purified and stored in MMG solution (Mannitol–MES–MgCl_2_) until ready for transfection experiments. (**4**) sgRNAs were either synthetically created which require crRNA and tracrRNA hybridization, or invitro transcribed with T7 containing sgRNA and tracrRNA DNA template. (**5**) Prior to transfection, Cas9 and sgRNA is assembled at RT. (**6**) Cas9 RNP’s are introduced to protoplast cells stored in MMG solution and incubated in selected PEG concentrations for transfection. (**7a**) Protoplast are washed and either co-cultivated with explants (nurse culture) for regeneration or (**7b**) DNA is isolated from pooled protoplast samples to perform mutation detection. (**8**) Mutation detection is performed by either the T7 mutation detection assay or by sequencing. Created in BioRender. Brown, A. (2026) https://BioRender.com/nyri57i.

Leaf protoplasts from *Ipomoea batatas* cultivar NZ196 (PI-318846, D-3) were isolated from four to six weeks old in vitro-grown plantlets (Figure 9). Following an in-house protocol [16]. Under aseptic conditions, leaves were sectioned into 0.1–1 mm strips in glass Petri plates containing a few drops of hypertonic plasmolysis solution (0.33 M Mannitol, 20 mM MES pH 5.7, 20 mM KCL, 10 mM CaCl_2_, and 0.1% BSA to prevent protoplasts from bursting. Samples were transferred into new 100 × 15 mm Petri plates, immersed in plasmolysis solution, and incubated in a desiccator for ~15 min to aid in separating protoplasts from cell walls [76]. The plasmolysis solution was then removed, and samples were immersed in 8–10 mL of cell wall digestion solution containing 20 mM MES, 0.5 M Mannitol, 0.6% Cellulase Onozuka R-10 (Yakult Honsha C., Ltd., Tokyo, Japan), 0.5% Macerozyme R-10 (Yakult Honsha C., Ltd., Japan), 0.1% Pectolyase Y23 (Yakult Honsha C., Ltd., Japan), 20 mM KCl, 10 mM CaCl_2_, and 0.1% BSA. Samples were vacuum infiltrated for ~30 min and incubated at room temperature for 7 h at 40 rpm on a rotary shaker (ORBITRON II Rotator model # 26025, Boekel Scientific, PA, USA) [16,77]. Protoplast cells were counted using a TC20 cell counter (Bio-Rad, Hercules, CA, USA, catalog # 145-010) and a hemocytometer. The digested sample was diluted with an equal volume of W5 solution (2 mM MES, 154 mM NaCl, 125 mM CaCl_2_, and 5 mM KCl) and filtered through a 40 µm nylon mesh into a 50 mL beaker. Released protoplasts were gently transferred into a 30 mL Oakridge tube and centrifuged at 200× *g* for 3 min to harvest. The supernatant was discarded, and protoplasts were resuspended in 3–5 mL W5 solution for an additional wash. Protoplasts were gently swirled and centrifuged at 150× *g* for 2 min, and the W5 solution was removed. Following purification, protoplasts were resuspended in MMG solution (Mannitol–MES–MgCl_2_) and adjusted to 1 × 10^5^ for CRISPR RNP transfection. Control samples were stored in a 1:1 mixture of W1 solution (4 mM MES, 0.4 M Mannitol, and 0.02 M KCl) and callus production (CP) media [73,78,79]. Unless otherwise specified, all reagents and supplies were purchased from MilliporeSigma (St. Louis, MO, USA).

### 3.5. CRISPR-Cas9 Editing of Sweetpotato Protoplast by PEG-Mediated RNPs Transfection

To improve site-specific mutagenesis efficiency in sweet-potato-targeting selected eukaryotic translation initiation factors, *IbeIF(iso)4E*, resuspended protoplasts were exposed to pre-assembled CRISPR-Cas9 RNP complexes via PEG-mediated transfection (Figure 9). Cas9, tracrRNAs, and synthetic gRNAs were synthesized by IDT-DNA (Coralville, IA). The sgRNAs utilized were synthesized by IDT as RNA-oligonucleotide molecules for RNP work, adjusted to desired ratios before RNP assembly. Prior to transfection, CRISPR components (Cas9 and sgRNA) were pre-assembled at RT for ~20 min. 250 µL of 1 × 10^5^ protoplast stored in MMG solution were aliquoted into 2 mL tubes using 200 µL orifice pipette tips and transfected with Cas9 and sgRNA ratios of 3:1 (45 µg:15 µg) and 1:3 (15 µg:45 µg) in a 20 µL reaction with 1× NEB buffer 3 (New England Biolabs, Ipswich, MA # B7003S), following [80]. Approximately 210 µL of PEG solution at Tr1 = 25% or Tr2 = 40% was added to separate tubes and incubated for 25 min at room temperature. After RNP assembly, Cas9 protein/sgRNA complex were added to the protoplasts, followed immediately by an equal volume of PEG, and the mixture was gently mixed. Next, 1 mL of W5 solution was added, and the mixture was incubated for an additional 10 min. Transfected protoplasts were pelleted at 100× *g* for 3 min, resuspended gently in W1:CP medium, and incubated in the dark for 36 to 48 h. Three biological replicates were performed. Two days after transfection, half of the samples were used for T7 mutation detection, while the remaining samples were subjected to previously established nurse-feeder somatic embryogenesis regeneration protocols [16].

### 3.6. Plant Regeneration from Sweetpotato CRISPR-RNP-Transfected Protoplast on Established Feeder-Nurse Leaf Explant

The Novel nurse-culture protocol system for sweetpotato protoplast regeneration established at Tuskegee University [16,78,81] and served as a conditioned feeder platform for protoplast regeneration. Concomitant with the post-transfection incubation period, the nurse-culture system was utilized by plating freshly excised sweetpotato D-3 leaf explants (0.5 × 0.5 in diameter) adaxial side-down onto sweetpotato callus production (CP) media based on [73,78,79,82], as modified by [48]. CPI media was supplemented with 0.25 mg/L BAP, 2.46 mg/L 2,4-D, 3% sucrose, adjusted to pH 5.8 with 0.3 M KOH, and solidified with 0.3% Phytagel. PEG-mediated CRISPR–Cas9–sgRNA RNP-transfected protoplasts were then gently overlaid (50 µL per explant) onto freshly excised and plated sweetpotato leaf explants, which served as a nurse-culture feeder layer. Large-orifice pipette tips were used to prevent mechanical damage to the intact protoplasts during transfer. For regeneration, protoplast-over-explant samples were incubated under light (50 μmol m^−2^ s^−1^) at 27 °C ± 1 °C for 5 days and then transferred to darkness for 10 days to promote cell division and elongation, and colony formation on the leaf explant support. After 2 weeks on CPI medium, co-cultivated samples were transferred to CPII medium (CP with 0.221 mg/L 2,4-D, 3% sucrose, pH 5.8, and 0.3% phytagel) and incubated in darkness at 27 °C ± 1 °C for one week to induce callus formation. Following a total of 3 weeks incubation on CPI and CPII media, with the plates sealed with parafilm, callus-explant tissues were transferred to embryo production medium (EP: MS with reduced NH_4_NO_3_ (800.4 mg/L) and KNO_3_ (1900 mg/L), supplemented with 2.5 mg/L ABA, pH 5.8, and 0.3% phytagel. Plates were sealed with 3 M micropore tape (MilliporeSigma, St. Louis, MO, USA) to maintain gas exchange and incubated at 27 °C ± 1 °C under a 14/10 h photoperiod (50 μmol m^−2^ s^−1^) until somatic embryos developed and plantlets regenerated. Developing embryos obtained after 4–6 weeks on EP medium were transferred to EP medium without ABA (EPO) for further maturation and germination. Control samples (with non-transfected protoplasts) were subjected to the same treatments. Additionally, the established somatic embryogenesis of sweetpotato leaf explants developed by [82] served as overall control without protoplast [48]. Putative edited germinated embryos were transferred to GA-7 vessels containing MM for further plantlet development. Genomic DNA from regenerated putative edited plantlets and controls were then subjected to mutation detection analyses using PCR-RE T7 assay from New England Biolabs, amplicon sequencing, and InDels mining.

### 3.7. Mutation Detection by PCR-RE and Targeted Sequencing and Mutation Efficiency

To detect CRISPR-induced indels at the *IbeIF(iso)4E* locus, PCR amplicons spanning the target region were analyzed using the T7 Endonuclease I (T7EI) mutation detection assay. After PCR amplification, products were denatured and reannealed to permit heteroduplex formation between wild-type and indel-containing alleles. T7EI cleaves mismatched or bulged regions generated by insertions or deletions, and the resulting fragments were resolved on agarose gels. Representative samples were subsequently subjected to Sanger sequencing to confirm the presence and identity of edited alleles. Genomic DNA was isolated from non-transfected protoplasts, CRISPR–Cas9 RNP-transfected protoplasts, and putatively edited regenerated plantlets using the GeneJET Plant Genomic DNA Purification Kit (Thermo Scientific, Waltham, MA, USA; #K0791). The target locus was amplified using Q5^®^ High-Fidelity 2× Master Mix from the EnGen^®^ Mutation Detection Kit (NEB, Ipswich, MA, USA; E3321) in 25 μL reactions. Primers (Table 5) were designed to generate ~600 bp amplicons spanning the *IbeIF(iso)4E* CRISPR cleavage site. Thermocycling conditions were 98 °C for 3 min; 35 cycles of 98 °C for 1 min, 58 °C for 1 min, and 72 °C for 1 min; followed by a 2 min extension at 72 °C. PCR products were stored at 4 °C prior to mutation analysis, and 5 μL of each reaction was first visualized on a 1% agarose gel to verify amplicon size. For T7EI digestion, WT and transfected PCR amplicons were mixed in equal volumes (2.5 μL each) to generate approximately 50% heteroduplex DNA. The mixture was combined with 2 μL 10× NEB Buffer 2 and 12 μL nuclease-free water and subjected to the following annealing program: 95 °C for 10 min; cooling from 95 °C to 85 °C at −2 °C/s; then from 85 °C to 25 °C at −0.1 °C/s. After heteroduplex formation, 1 μL T7 Endonuclease I was added, and samples were incubated at 37 °C for 30 min. T7EI was then inactivated by adding 1 μL Proteinase K and incubating at 37 °C for 5 min. Digestion products were separated on 2% agarose gels stained with ethidium bromide, and mutation frequencies were quantified using ImageJ software [83]. Regenerated plantlets were evaluated directly by PCR-RE analysis without heteroduplex mixing. For further validation, PCR amplicons were submitted for Sanger sequencing (Azenta–GENEWIZ, South Plainfield, NJ, USA), and CRISPR editing efficiency was calculated using Inference of CRISPR Edits (ICE). InDelS percentages were quantified based on insertions, deletions, and substitutions detected within the CRISPR–Cas9 cleavage sites [60].

### 3.8. Identification of Sweetpotato Off-Target Site

Potential off-target sites were assessed by aligning the protospacer sequence of *IbeIF(iso)4E*, both with and without the adjacent PAM sequence, against two diploid sweetpotato reference genomes: *Ipomoea trifida* (NSP306) and *Ipomoea triloba* (NSP323), available through the Sweetpotato Genomics Resource. No sequences exhibiting sufficient homology to the target site were identified, indicating a low likelihood of off-target activity for the selected sgRNA.

## 4. Conclusions

This study successfully optimized site-specific genome editing in sweetpotato by leveraging CRISPR/Cas9 RNP technology and PEG-mediated protoplast transfection, targeting the *IbeIF(iso)4E* gene in the highly regenerative cultivar PI-318846 (PI-318846-3/N.Z.196). We demonstrated that using 25% PEG in combination with 15 µg Cas9 and 45 µg sgRNA for 25 min of RNP transfection post-72 h resulted in considerable targeted mutagenesis frequencies, consistent with prior observations that mutation efficiency is dependent on gRNA sequence, exposure time, and optimal Cas9:sgRNA ratios [19,40,84]. Sequencing and T7 mutation detection revealed indels near the sgRNA target site in three independent edited plantlets; one from the 3:1 ratio and two from the 1:3 ratio—yielding an overall mutation efficiency of 3.1% from 96 explants. The resulting frameshift mutations introduced premature stop codons in the IbeIF(iso)4E protein, confirming successful site-directed mutagenesis. Both RNP ratios generated edits in similar deletion sites, with the 1:3 Cas9:sgRNA ratio producing the highest number of mutations. This work outlines a complete workflow for CRISPR/Cas9–sgRNA RNP editing in sweetpotato, including gRNA design, T7-based in vitro sgRNA synthesis, RNP preassembly, PEG-mediated transfection, plantlet regeneration, and mutation screening (Figure 6, Figure 7 and Figure 8). To our knowledge, this is the first report demonstrating a positive correlation between RNP amount combined with PEG and site-directed mutagenesis efficiency in plants, and it establishes that the entire process can be completed within 12–18 weeks using the highly regenerative sweetpotato cultivar PI-318846.

## Figures and Tables

**Table 1:** Predicted secondary and tertiary structure of each *eIF4E* gene.

Predicted Protein	α-Helices	β-Pleated Sheets	Protein Size kDa
IbeIF4E	9	8	26.04
IbeIF(iso)4E	6	8	22
IbCBP	6	8	25

**Table 2:** List of gRNAs designed to target sweetpotato eIF4 family within this study.

	Sequence (5′-3′)
Single Guide RNA	*IbeIF4E*
sgRNA1	TCATCAGCTAGGCCCAGCGA
sgRNA2	AGAAGTCGTCATCATCAGCT
sgRNA3 *	TATACGTTGTTGGCAATGAT
sgRNA4 *	ATTGGAGAACAATTTGACCA
	*IbeIF(iso)4E*
sgRNA1	GACGGCAGCTGAATTGACGG
sgRNA2	GAAGATCCTGAGTGTGCCAA
sgRNA3 *	GCTTGTATGATCAGATATTT
sgRNA4 *	GGAGCAATTTGATGAAGCAG
sgRNA5	GTGTGCGTCAGAGACAGGAC
	*IbCBP*
sgRNA1	TCAGCCGCCGACCTCTCTGA
sgRNA2	ATGTGTTTTGGTATACTCGC
sgRNA3 *	ATGGATTTCAGTACAGTTGA
sgRNA4 *	TGGTATACTCGCCGGACTCC

The numbers (1−5) indicate distinct single-guide RNA sequences designed to target different regions of the genes. The guide with the (*) were further synthesized as RNA oligonucleotides for RNP.

**Table 3:** Embryogenic potential hexaploid sweetpotato form PEG-mediated CRISPR/cas9 RNP-transfected sweetpotato into regenerable edited PI-318846 plantlets.

	EMBRYOGENIC RESPONSE (%)				
Treatment	25% PEG	40% PEG	Mean Number of Embryos/Explant	Number of Regenerated Edited Plants	Mutation Frequency
eIF(iso)4EG4 sgRNA only	83.3%	0	3.3 ± 0.3	-	-
eIF(iso)4EG4 Cas only	91.6%	0	3.6 ± 0.3	-	-
eIF(iso)4EG4 3:1	75%	0	3 ± 0.57	6	16%
eIF(iso)4EG4 1:3	66.6%	0	2.6 ± 0.6	4	50%
Non-transfected Normal somatic embryogenesis	- 91.6%	-	3.6 ± 0.3	-	-

**Table 4:** List of primers designed to transcribe sgRNA to perform in vitro transcription sgRNA cleavage assay.

Primer	Sequence (5′-3′)
	*IbeIF4E*
sgRNA1	TAATACGACTCACTATAGGTCATCAGCTAGGCCCAGCGAGTTTAAGAGCTATGC
sgRNA2	TAATACGACTCACTATA GG AGAAGTCGTCATCATCAGCT GTTTAAGAGCTATGC
sgRNA3	TAATACGACTCACTATA GG TATACGTTGTTGGCAATGAT GTTTAAGAGCTATGC
sgRNA4	TAATACGACTCACTATA GG ATTGGAGAACAATTTGACCA GTTTAAGAGCTATGC
	*IbeIF(iso)4E*
sgRNA1	TAATACGACTCACTATA G GACGGCAGCTGAATTGACGG GTTTAAGAGCTATGC
sgRNA2	TAATACGACTCACTATA G GAAGATCCTGAGTGTGCCAA GTTTAAGAGCTATGC
sgRNA3	TAATACGACTCACTATA G GCTTGTATGATCAGATATTT GTTTAAGAGCTATGC
sgRNA4	TAATACGACTCACTATA GGAGCAATTTGATGAAGCAG GTTTAAGAGCTATGC
sgRNA5	TAATACGACTCACTATAGGTGTGCGTCAGAGACAGGACGTTTAAGAGCTATGC
	*IbCBP*
sgRNA1	TAATACGACTCACTATAGGTCAGCCGCCGACCTCTCTGAGTTTAAGAGCTATGC
sgRNA2	TAATACGACTCACTATAGGATGTGTTTTGGTATACTCGCGTTTAAGAGCTATGC
sgRNA3	TAATACGACTCACTATAGGATGGATTTCAGTACAGTTGAGTTTAAGAGCTATGC
sgRNA4	TAATACGACTCACTATAGGTGGTATACTCGCCGGACTCCGTTTAAGAGCTATGC

Sequences indicated in green encode T7 protomer site. Guanine nucleotides indicated in red were incorporated to enhance T7 activity. Sequences without highlighted G nucleotides, already contained Guanine nucleotides at the 5′ sgRNA site. Nucleotides indicated in black encode for pre-designed sgRNAs, and sequences indicated in pink encode for the 5′ site of scaffold (tracrRNA) sequences. The numbers (1−5) indicate distinct single-guide RNA sequences designed to target different regions of the genes.

**Table 5:** List of primers designed to amplify target DNA/cDNA template for in vitro transcription sgRNA cleavage assay.

Primer Name	Sequence (5′-3′)	Expected Amplicon Size (gDNA/cDNA)
*Ib*eIF4E-FL-FR	ATGGTGGAAGAAATCGAGAAATCG	2440 bp/696 bp
*Ib*eIF4E-FL-RV	TACTGTGTAACGATTCTTGGC	
eFI4E 2.3-FR	GTGTTTACCACGCAACATTGAT	600 bp/-
eIF4E 2.3-RV	ACAAGCATCTTAATTGCGACTTG	
*Ib*eIF(iso)4E-FL-FR	ATGGCAACCGGAGACGGCAG	2316 bp/606 bp
*Ib*eIF(iso)4E-FL-RV	CACACTATAACGCCCCTTAGCTG	
eIF(iso)4E 2.3-FR	CTGTTGCAAGCATGATGTTATGT	612 bp/-
eIF(iso)4E 2.3-RV	GGAATGCACAGAGGCAGTAG	
*Ib*CBP-FL-FR	ATGGAAGAAGCGATAGCAGAG	2585 bp/675 bp
*Ib*CBP-FL-RV	GCCTCTCAACCAAGTGTTCCG	
CBP 2.4-FR	GTGATTGTGAGCAGTGAATGAAC	609 bp/-
CBP2.4-RV	GGACGAATTCCCTCCTTGAAA	

## Data Availability

The original contributions presented in this study are included in the article. Further inquiries can be directed to the corresponding author.

## Abbreviations

ABA	Abscisic acid
BAP	6-Benzylaminopurine
BSA	Bovine serum albumin
bp	Base pair
CBP	Cap-binding protein
CDS	Coding sequence
Cas9	CRISPR-associated protein 9
CP	Callus production medium
CRISPR	Clustered regularly interspaced short palindromic repeats
crRNA	CRISPR RNA
cDNA	Complementary DNA
DSB	Double-strand break
eIF4E	Eukaryotic translation initiation factor 4E
eIF(iso)4E	Isoform of eukaryotic translation initiation factor 4E
EP	Embryo production medium
gDNA	Genomic DNA
gRNA	Guide RNA
Ib	*Ipomoea batatas*
*IbCBP*	*Ipomoea batatas* cap-binding protein gene
*IbeIF4E*	*Ipomoea batatas eIF4E* gene
*IbeIF(iso)4E*	*Ipomoea batatas eIF(iso)4E* gene
ICE	Inference of CRISPR Edits
Indel	Insertion/deletion mutation
MES	2-(N-morpholino)ethanesulfonic acid
MM	Multiplication medium
MMG	Mannitol–MES–MgCl_2_ solution
MS	Murashige and Skoog medium
NHEJ	Non-homologous end joining
PAM	Protospacer adjacent motif
PEG	Polyethylene glycol
PCR	Polymerase chain reaction
PCR-RE	PCR-restriction enzyme assay
RNP	Ribonucleoprotein
RT	Room temperature
sgRNA	Single guide RNA
SNP	Single-nucleotide polymorphism
SPFMV	Sweetpotato feathery mottle virus
SPCSV	Sweetpotato chlorotic stunt virus
SPVD	Sweetpotato virus disease
T7EI	T7 endonuclease I
WT	Wild type
W5	Protoplast wash solution
